# Supplementary material for: A LAT-Based Signaling Complex in the Immunological Synapse as Determined with Live Cell Imaging Is Less Stable in T Cells with Regulatory Capability
Source: Cells. 2021 Feb 17;10(2):418. doi: 10.3390/cells10020418 (PMC7921939; doi:10.3390/cells10020418)
Supplement: Supplementary file 1 [file cells-10-00418-s001.zip › supplement/cells-1042866-supplementary-captions.docx]

Early signaling is terminated more rapidly in T cells with regulatory capability

Yikui Li ^1,†^, Helen M. Tunbridge ^1,†^, Graham J. Britton ^1, 2^, Elaine V. Hill ^1^, Parisa Sinai ^1^, Silvia Cirillo ^1^, Clare Thompson ^3^, Farnaz Fallah-Arani ^3^, Simon J. Dovedi ^4^, David C. Wraith ^1,5,*^ and Christoph Wülfing ^1,*^

1 School of Cellular and Molecular Medicine, University of Bristol, Bristol, BS8 1TD, United Kingdom

2 Precision Immunology Institute, Icahn School of Medicine at Mount Sinai, New York, NY 10029, USA

3 Immunology Therapeutic Area, UCB, Slough, SL1 3WE, United Kingdom

4 R&D Oncology, AstraZeneca, Granta Park, Cambridge, CB21 6GH, United Kingdom

5 Institute of Immunology and Immunotherapy, University of Birmingham, Birmingham, B15 2TT, United Kingdom

***** Correspondence: [Christoph.Wuelfing@bristol.ac.uk](mailto:Christoph.Wuelfing@bristol.ac.uk) (C.W.), [d.wraith@bham.ac.uk](mailto:d.wraith@bham.ac.uk) (D.C.W.)

† these authors contributed equally to this work

**Supplementary Figure S1. UCB9608 enhances FoxP3 expression.** Tg4 splenocytes were incubated with 1 µg/ml α-CD3 plus 2 µg/ml α-CD28 for 5 days in the presence of 10 ng/ml TGFβ plus 100 U/ml IL-2 with the indicated concentration of UCB9608 or vehicle and stained for FoxP3. Data are expressed as MFI of FoxP3^+^. One representative experiment of 2. * p<0.05, ** p<0.01.

**Supplementary Figure S2. LAT association with the cSMAC is terminated more rapidly in T cells with regulatory capability. A** The panel graphically represents the six categories used to classify spatiotemporal sensor distribution as underpinned by defined cell biological structures at the T cell:APC interface [21, 59]. The antigen-presenting cell above the T cell is not shown. Central reflects the cSMAC, lamellal an F-actin-based lamella extending from the undulating T cell plasma membrane deep into the T cell, peripheral the part of the actin network stabilizing the interface edge. Diffuse reflects cortical accumulation, invagination enrichment in a transient large T cell invagination and asymmetric individual small lamellae. **B,C** Purified CD4^+^ Tg4 T cells were activated with 1 µg/ml α-CD3 plus 2 µg/ml α-CD28 in the presence of 50 U/ml IL-2 or 10 ng/ml TGFβ plus 100 U/ml IL-2. B Schematic comparison to activation of Tg4 splenocytes with MBP Ac1-9[4K] peptide. C Data are expressed as % FoxP3^+^ cell at day 5 of tissue culture. Tg4 iTreg pep data are from Figure 1C. **D,E** LA Tg4 iTreg or Teff cells transduced to express LAT-GFP as indicated were activated with PL8 APCs (10 µg/ml Ac1-9[4Y]). D Data are expressed as the percentage of cell couples with LAT-GFP accumulation in the indicated patterns (Supplementary Figure S2A) relative to tight cell couple formation. Number of cell couples analyzed are n=42, 68 for Tg4 Teff ab, iTreg ab cells, respectively, from 2 and 5 independent experiments. Teff pep and iTreg pep data are from Figure 2B. Statistical significance of differences between conditions is given in Table S2. E The same data are expressed as the log_2_ of the ratio of % Tg4 T cells with accumulation in the central over the invagination pattern relative to tight cell couple formation Teff pep and iTreg pep data are from Figure 2C. Data points are slightly nudged to increase legibility. **p<0.01 vs. Teff ab.

**Supplementary Figure S3. Computational analysis of LAT accumulation in Tg4 Teff pep and iTreg pep cells.** Computational analysis of the same Tg4 Teff pep and iTreg pep LAT-GFP data as in Figure 2B. **A** Population-averaged models of LAT-GFP accumulation in Tg4 Teff pep and iTreg pep cells as indicated are given relative to tight cell couple formation in sequential z sections through the 3D model in a rainbow style false color scale. **B** On top, population and time-averaged models of LAT-GFP accumulation in Tg4 Teff pep and iTreg pep cells as indicated are given as in A. Below the 10% of the cell volume with the highest LAT-GFP intensity is given in yellow. Data are expressed at the bottom as enrichment in the 10% of the cell volume with the highest LAT-GFP intensity relative to the rest of the cell relative to tight cell couple formation. **C** On top, population and time-averaged models of LAT-GFP accumulation in Tg4 Teff pep and iTreg pep cells as indicated are given the same as in B. Below a cylinder representing the interface center is given in yellow. Data are expressed at the bottom as enrichment in the central interface cylinder relative to the rest of the cell relative to tight cell couple formation.

**Supplementary Figure S4. cSMAC formation was diminished but not abolished upon induction of a regulatory phenotype.** T cells as indicated transduced to express TCRζ-GFP were activated with PL8 APCs (10 µg/ml Ac1-9[4Y]). Data are expressed as the percentage of cell couples with TCRζ-GFP accumulation in the indicated patterns (Supplementary Figure S2A) relative to tight cell couple formation. Number of cell couples analyzed is n=50 for Tg4 iTreg ab cells from 3 independent experiments. iTreg pep data are from Figure 3C. Statistical significance of differences between conditions is given in Table S4.

**Supplementary Figure S5.** Actin-driven formation of a polarized cell couple is impaired upon induction of a regulatory phenotype. A Tg4 T cells as indicated transduced to express F-tractin-GFP were activated with PL8 APCs (10 µg/ml Ac1-9[4Y]). Data are expressed as the percentage of cell couples with F-tractin-GFP accumulation in the indicated patterns (Supplementary Figure S2A) relative to tight cell couple formation. Number of cell couples analyzed is n=48 for Tg4 iTreg ab cells from 3 independent experiments. iTreg pep data are from Figure 4B. Statistical significance of differences between conditions is given in Table S6. B-D Tg4 T cells as indicated transduced to express LAT-GFP were activated with PL8 APCs (10 µg/ml Ac1-9[4Y]). These are the same cells as in Fig. 2B. B The scheme indicates the way morphology measurements were taken. C On the left, data are expressed as the ratio of interface to cell diameter at the indicated time relative to tight cell coupling; in the middle, as the ratio of lamellal to cell length; on the right, as the T cell shape factor (ratio of lamellal length to interface diameter). D The same data are expressed as the percentage of Tg4 T cells with a distinct cell-wide lamellal sheet that the T cell uses to make contact with the APC at the indicated time relative to tight cell coupling.

**Supplementary Figure S6. cSMAC formation can be partially restored with synthetic approaches in Tg4 iTreg pep cells. A** Tg4 iTreg cells as indicated transduced to express LAT V3-GFP were activated with PL8 APCs (10 µg/ml Ac1-9[4Y]). Data are expressed as the percentage of cell couples with LAT V3-GFP accumulation in the indicated patterns (Supplementary Figure S2A) relative to tight cell couple formation. Number of cell couples analyzed is n=59 for Tg4 iTreg ab cells from 4 independent experiments. iTreg pep data are from Figure 6B. Statistical significance of differences between conditions is given in Table S9. **B** Computational image analysis similar to Supplementary Figure S3 of the same data as in Figure 6B. On the left, data are expressed as enrichment in the 10% of the cell volume with the highest LAT-GFP, LAT-V3-GFP or LAT Vav-GFP intensity relative to the rest of the cell relative to tight cell couple formation. On the right, data are expressed as enrichment in the central interface cylinder relative to the rest of the cell relative to tight cell couple formation as indicated. LAT-GFP data are from Supplementary Figure S3. **C** Similar to Supplementary Figure S5B right, the same data as in Fig. 6B are expressed as the T cell shape factor (ratio of lamellal length to interface diameter) at the indicated time relative to tight cell coupling. LAT-GFP data are from Supplementary Figure S5B.

**Supplementary Figure S7. Pd-1 and Ctla-4 impair cSMAC formation upon induction of a regulatory phenotype. A** Tg4 T cells as indicated transduced to express LAT-GFP were activated with PL8 APCs (10 µg/ml Ac1-9[4Y]). Data are expressed as the percentage of cell couples with LAT-GFP accumulation in the indicated patterns (Supplementary Figure S2A) relative to tight cell couple formation. Number of cell couples analyzed are n=43, 49, 37 for Tg4 iTreg ab cells in the presence of 10 µg/ml α-Pd-1, 10 µg/ml α-Ctla-4 and 10 µg/ml α-Pd-1 plus α-Ctla-4, respectively, from **n-n** independent experiments each. Statistical significance of differences between conditions is given in Table S11. Tg4 iTreg ab buffer only data are from Supplementary Figure S2D. **B** Tg4 iTreg ab cells transduced to express TCRζ-GFP were activated with PL8 APCs (10 µg/ml Ac1-9[4Y]) in the presence of 10 µg/ml α-Pd-1 plus α-Ctla-4. Data are expressed as percentage of cell couples with TCRζ-GFP accumulation in the indicated patterns (Supplementary Figure S2A) relative to tight cell couple formation. Number of cell couples analyzed are n=59 from **n** independent experiments. Statistical significance of differences between conditions is given in Table S11. **C** Tg4 T cells as indicated transduced to express F-tractin-GFP were activated with PL8 APCs (10 µg/ml Ac1-9[4Y]) in the presence of 10 µg/ml α-Pd-1 plus α-Ctla-4. Data are expressed as percentage of cell couples with F-tractin-GFP accumulation in the indicated patterns (Supplementary Figure S2A) relative to tight cell couple formation. Number of cell couples analyzed are n=37 from **n** independent experiments. Statistical significance of differences between conditions is given in Table S11. **D** Tg4 T cells as indicated transduced to express LAT-GFP were activated with PL8 APCs (10 µg/ml Ac1-9[4Y]). These are the same cells as in Figure 7B. Similar to Supplementary Figure S5B right, data are expressed as the T cell shape factor (ratio of lamellal length to interface diameter) at the indicated time relative to tight cell coupling. Tg4 Teff pep and iTreg pep data are from Supplementary Figure S5B. * p<0.05, *** p<0.001, **** p<0.0001 vs. iTreg pep at the same time. **E, F** Tg4 splenocytes were incubated with 10 µg/ml MBP Ac1-9[4K] peptide for 6 days in the presence of 10 ng/ml TGFβ plus 100 U/ml IL-2 upon addition of 10 µg/ml α-Pd-1, 10 µg/ml α-Ctla-4, 10 µg/ml α-Pd-1 plus α-Ctla-4 or buffer only. E Data are expressed as the percentage FoxP3^+^ cells (n=4 independent experiments). * p<0.05, ** p<0.01 vs. buffer only on the same day. F Data from a larger number of experiments including those in E are expressed as the percentage FoxP3^+^ cells on day 6 only. The part of the data without antibody treatment are the same as Figure 1C. (n=12, 12 independent experiments). **** p<0.0001. **G** Tg4 splenocytes were incubated with 10 µg/ml MBP Ac1-9[4K] peptide for 6 days in the presence of 10 ng/ml TGFβ plus 100 U/ml IL-2 upon addition of 10 µg/ml α-Pd-1 plus α-Ctla-4 or buffer only and IL-10 amounts in tissue culture supernatants were determined by ELISA. Data are expressed as amount of cytokine (n=4-5 independent experiments). Data points are slightly nudged to increase legibility. The difference between α-Pd-1 plus α-Ctla-4 and buffer only is significant with p<0.01 by 2-way ANOVA.

**Supplementary Figure S8. Computational analysis of LAT accumulation in iTreg pep cells upon blockade of Pd-1 and Ctla-4.** Computational image analysis similar to Supplementary Figure S3 of the same data as in Figure 7B. On the left, data are expressed as enrichment in the 10% of the cell volume with the highest LAT-GFP intensity relative to the rest of the cell. On the right, data are expressed as enrichment in the central interface cylinder relative to the rest of the cell relative to tight cell couple formation. Tg4 Teff pep and iTreg pep data are from Supplementary Figure S3.

**Video S1.** A representative interaction of a Tg4 T eff pep cell retrovirally transduced to express LAT-GFP with a PL8 B cell lymphoma APCs and 10 μg/ml MBP Ac1-9[4Y] peptide is shown. DIC images are shown on the top, with matching top-down, maximum projections of 3D sensor fluorescence data on the bottom. The sensor fluorescence intensity is displayed in a rainbow-like, false-color scale (increasing from blue to red). 20 s intervals in video acquisition are played back as 2 frames per second. Cell coupling occurs in frame 4 (2s indicated video time).

**Video S2.** The video is displayed similar to Video S1. A Tg4 Ttol cell is transduced to express LAT-GFP and activated as in Video S1. Cell coupling occurs in frame 3 (1s indicated video time).

**Video S3.** The video is displayed similar to Video S1. A Tg4 iTreg cell is transduced to express LAT-GFP and activated as in Video S1. Cell coupling occurs in frame 5 (2s indicated video time).

**Video S4.** The video is displayed similar to Video S1. A Tg4 T eff pep 9608 cell is transduced to express LAT-GFP and activated as in Video S1. Cell coupling occurs in frame 7 (3s indicated video time).

**Video S5.** The video is displayed similar to Video S1. A Tg4 T eff pep cell is transduced to express tandem C1 domain-GFP and activated as in Video S1. Cell coupling occurs in frame 6 (3s indicated video time).

**Video S6.** The video is displayed similar to Video S1. A Tg4 T eff pep cell is transduced to express TCRζ-GFP and activated as in Video S1. Cell coupling occurs in frame 6 (3s indicated video time).

**Video S7.** The video is displayed similar to Video S1. A Tg4 T eff pep cell is transduced to express F-tractin-GFP and activated as in Video S1. Cell coupling occurs in frame 7 (3s indicated video time).

**Video S8.** The video is displayed similar to Video S1. A Tg4 iTreg cell is transduced to express LAT V3-GFP and activated as in Video S1. Cell coupling occurs in frame 7 (3s indicated video time).

**Video S9.** The video is displayed similar to Video S1. A Tg4 iTreg cell is transduced to express LAT-GFP and activated as in Video S1 upon addition of 10 µg/ml α-Pd-1 plus α-Ctla-4. Cell coupling occurs in frame 8 (4s indicated video time).
