# Supplementary material for: A LAT-Based Signaling Complex in the Immunological Synapse as Determined with Live Cell Imaging Is Less Stable in T Cells with Regulatory Capability
Source: Cells. 2021 Feb 17;10(2):418. doi: 10.3390/cells10020418 (PMC7921939; doi:10.3390/cells10020418)
Supplement: Supplementary file 1 [file cells-10-00418-s001.zip › supplement/cells-1042866-supplementary.pdf]

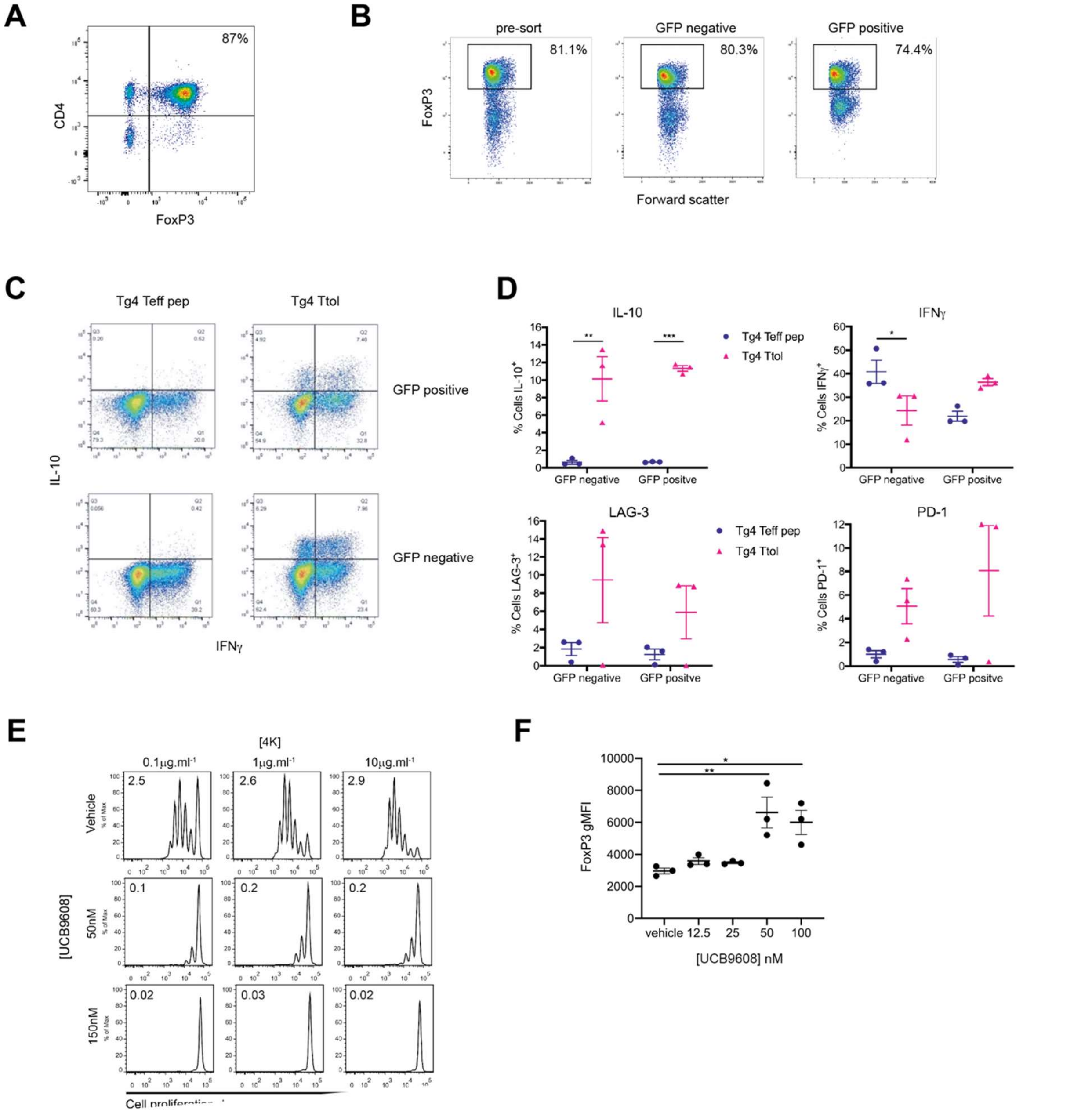

Figure S1. Three experimental models of T cells with regulatory capability. **A** Tg4 splenocytes were incubated with 10 μg/ml MBP Ac1-9[4K] peptide for 5 days in the presence of 50 U/ml IL-2 or 10 ng/ml TGFβ and 100 U/ml IL-2 and stained for CD4 and FoxP3. One representative FACS plot of 12 for incubation with 10 ng/ml TGFβ and 100 U/ml IL-2. **B** Tg4 splenocytes were incubated with 10 μg/ml MBP Ac1-9[4K] peptide for 5 days in the presence of 10 ng/ml TGFβ and 100 U/ml IL-2 and retrovirally transduced to express LAT-GFP, sorted into GFP^{+} and GFP^{-} cells and stained for FoxP3. One representative group of FACS plots of 2. **C** Tg4 T cells were tolerized *in vivo* with a dose escalation of MBP Ac1-9[4K] peptide in PBS or left untreated, incubated *in vitro* with 10 μg/ml MBP Ac1-9[4K] peptide for 5 days in the presence of 50 U/ml IL-2, retrovirally transduced to express GFP, re-activated with PMA and Ionomycin and stained for LAG-3, PD-1, intracellular IL-10 and IFNγ. One representative FACS plot of 3. **D** Tg4 T cells were tolerized *in vivo* with a dose escalation of MBP Ac1-9[4K] peptide in PBS or left untreated, incubated *in vitro* with 10 μg/ml MBP Ac1-9[4K] peptide for 5 days in the presence of 50 U/ml IL-2, retrovirally transduced to express GFP, re-activated with PMA and Ionomycin and stained for LAG-3, PD-1, intracellular IL-10 and IFNg. Representative FACS blots are given in C. Data are expressed as % T cells positive for the indicated marker for Ttol cells expressing LAT-GFP ('GFP-positive') or not ('GFP-negative')(n=3). The data for LAT-GFP-expressing Ttol cells are the same as Figure 1E. * $p<0.05$, ** $p<0.01$, *** $p<0.001$ **E** Naïve Tg4 T cells were labelled with the cell proliferation dye CellTrace Violet and incubated with irradiated splenocytes and the indicated concentrations of MBP Ac1-9[4K] peptide for 4 days in the presence of 50 U/ml IL-2 with the indicated concentration of UCB9608 or vehicle. One representative FACS plot of 3. **F** Tg4 splenocytes were incubated with 1 μg/ml α-CD3 plus 2

µg/ml α-CD28 for 5 days in the presence of 10 ng/ml TGFβ plus 100 U/ml IL-2 with the indicated concentration of UCB9608 or vehicle and stained for FoxP3. Data are expressed as MFI of FoxP3+. One representative experiment of 2. * $p<0.05$, ** $p<0.01$.

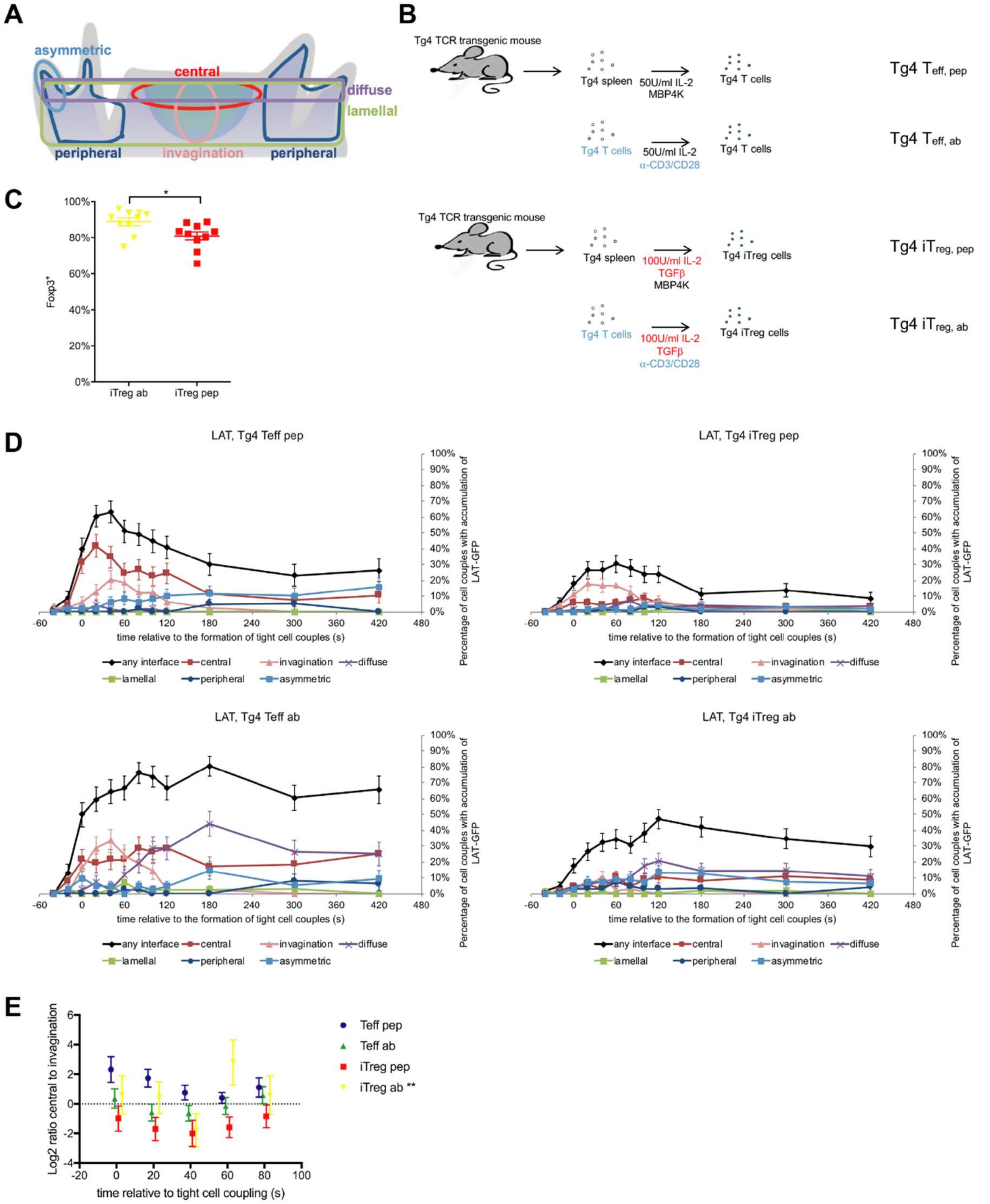

Figure S2. LAT association with the cSMAC is terminated more rapidly in T cells with regulatory capability. **A** The panel graphically represents the six categories used to classify spatiotemporal sensor distribution as underpinned by defined cell biological structures at the T cell:APC interface [21, 60]. The antigen-presenting cell above the T cell is not shown. Central reflects the cSMAC, lamellal an F-actin-based lamella extending from the undulating T cell plasma membrane deep into the T cell, peripheral the part of the actin network stabilizing the interface edge. Diffuse reflects cortical accumulation, invagination enrichment in a transient large T cell invagination and asymmetric individual small lamellae. **B,C** Purified CD4+ Tg4 T cells were activated with 1 µg/ml α-CD3 plus 2 µg/ml α-CD28 in the presence of 50 U/ml IL-2 or 10 ng/ml TGFβ plus 100 U/ml IL-2. B Schematic comparison to activation of Tg4 splenocytes with MBP Ac1-9[4K] peptide. C Data are expressed as % FoxP3+ cell at day 5 of tissue culture. iTreg pep data are from Figure 1B. **D,E** LA Tg4 iTreg or Teff

cells transduced to express LAT-GFP as indicated were activated with PL8 APCs (10 µg/ml Ac1-9[4Y]). D Data are expressed as the percentage of cell couples with LAT-GFP accumulation in the indicated patterns (Supplementary Figure S2A) relative to tight cell couple formation. Number of cell couples analyzed are n=42, 68 for Teff ab, iTreg ab cells, respectively, from 2 and 5 independent experiments. Teff pep and iTreg pep data are from Figure 2B. Statistical significance of differences between conditions is given in Table S2. E The same data are expressed as the $\log_2$ of the ratio of % Tg4 T cells with accumulation in the central over the invagination pattern relative to tight cell couple formation Teff pep and iTreg pep data are from Figure 2D. Data points are slightly nudged to increase legibility. **p<0.01 vs. Teff ab.

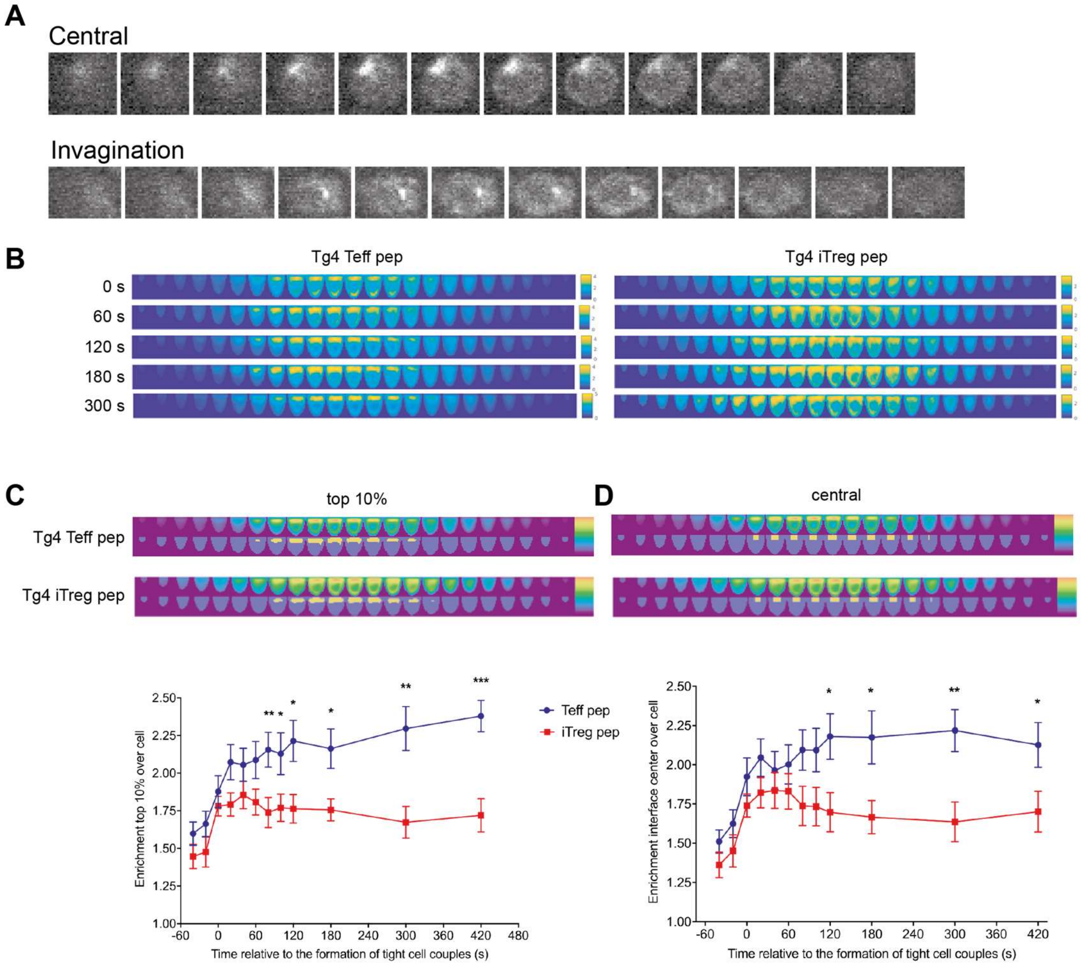

Figure S3. Representative imaging data and computational analysis of LAT accumulation in Tg4 Teff pep and iTreg pep cells. **A** Representative imaging data. Given are z-slices of the three-dimensional GFP fluorescence data of Tg4 Teff pep cells expressing LAT-GFP as bound to an APC (top left, right) with central and invagination accumulation as indicated. **B-D** Computational analysis of the same Teff pep and iTreg pep LAT-GFP data as in Figure 2B. B Population-averaged models of LAT-GFP accumulation in Teff pep and iTreg pep cells as indicated are given relative to tight cell couple formation in sequential z sections through the 3D model in a rainbow style false color scale. C On top, population and time-averaged models of LAT-GFP accumulation in Teff pep and iTreg pep cells as indicated are given as in A. Below the 10% of the cell volume with the highest LAT-GFP intensity is given in yellow. Data are expressed at the bottom as enrichment in the 10% of the cell volume with the highest LAT-GFP intensity relative to the rest of the cell relative to tight cell couple formation. D On top, population and time-averaged models of LAT-GFP accumulation in Teff pep and iTreg pep cells as indicated are given the same as in B. Below a cylinder representing the interface center is given in yellow. Data are expressed at the bottom as enrichment in the central interface cylinder relative to the rest of the cell relative to tight cell couple formation.

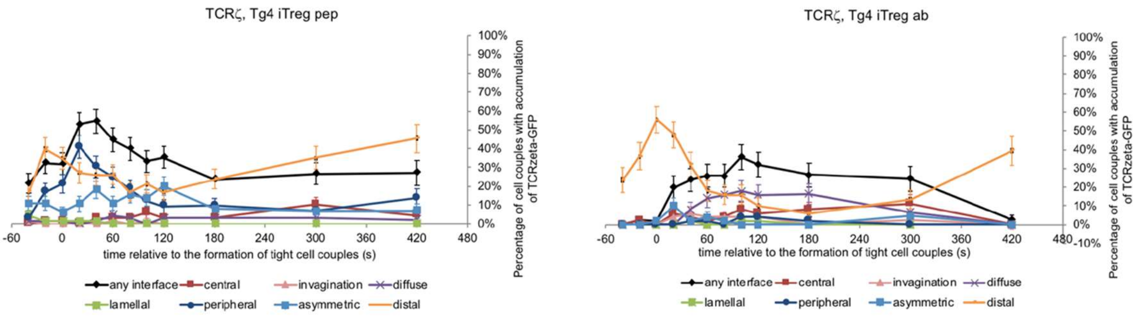

Figure S4. cSMAC formation was diminished but not abolished upon induction of a regulatory phenotype. Tg4 T cells as indicated transduced to express TCRζ-GFP were activated with PL8 APCs (10 µg/ml Ac1-9[4Y]). Data are expressed as the percentage of cell couples with TCRζ -GFP accumulation in the indicated patterns (Supplementary Figure S2A) relative to tight cell couple formation. Number of cell couples analyzed is n=50 for iTreg ab cells from 3 independent experiments. iTreg pep data are from Figure 3B. Statistical significance of differences between conditions is given in Table S4.

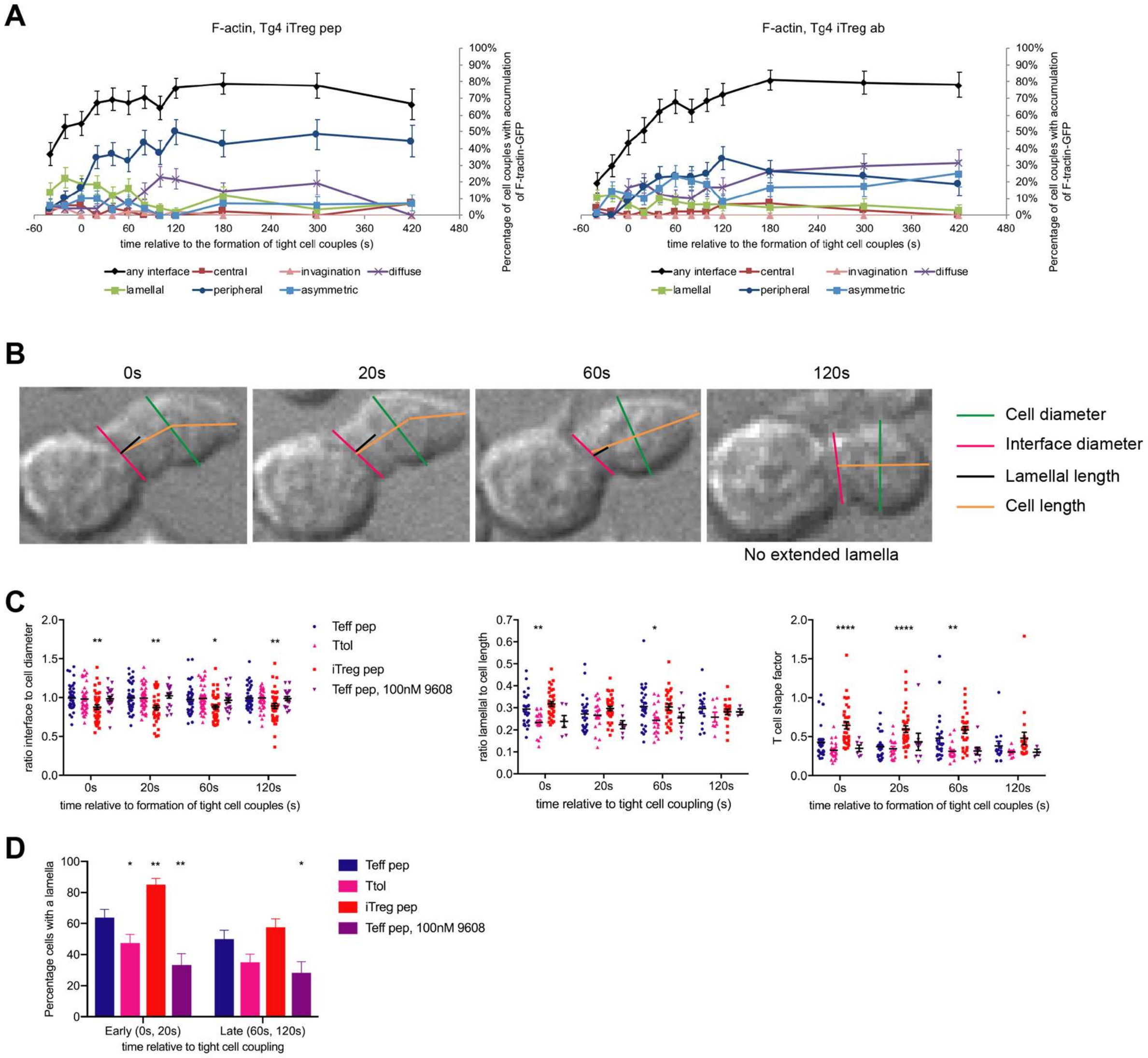

Figure S5. Actin-driven formation of a polarized cell couple is impaired upon induction of a regulatory phenotype. **A** Tg4 T cells as indicated transduced to express F-tractin-GFP were activated with PL8 APCs (10 µg/ml Ac1-9[4Y]). Data are expressed as the percentage of cell couples with F-tractin-GFP accumulation in the indicated patterns (Supplementary Figure S2A) relative to tight cell couple formation. Number of cell couples analyzed is n=48 for iTreg ab cells from 3 independent experiments. iTreg pep data are from Figure 5B. Statistical significance of differences between conditions is given in Table S6. **B-D** Tg4 T cells as indicated transduced to express LAT-GFP were activated with PL8 APCs (10 µg/ml

Ac1-9[4Y]). These are the same cells as in Fig. 2B. B The scheme indicates the way morphology measurements were taken. C On the left, data are expressed as the ratio of interface to cell diameter at the indicated time relative to tight cell coupling; in the middle, as the ratio of lamellal to cell length; on the right, as the T cell shape factor (ratio of lamellal length to interface diameter). D The same data are expressed as the percentage of Tg4 T cells with a distinct cell-wide lamellal sheet that the T cell uses to make contact with the APC at the indicated time relative to tight cell coupling.

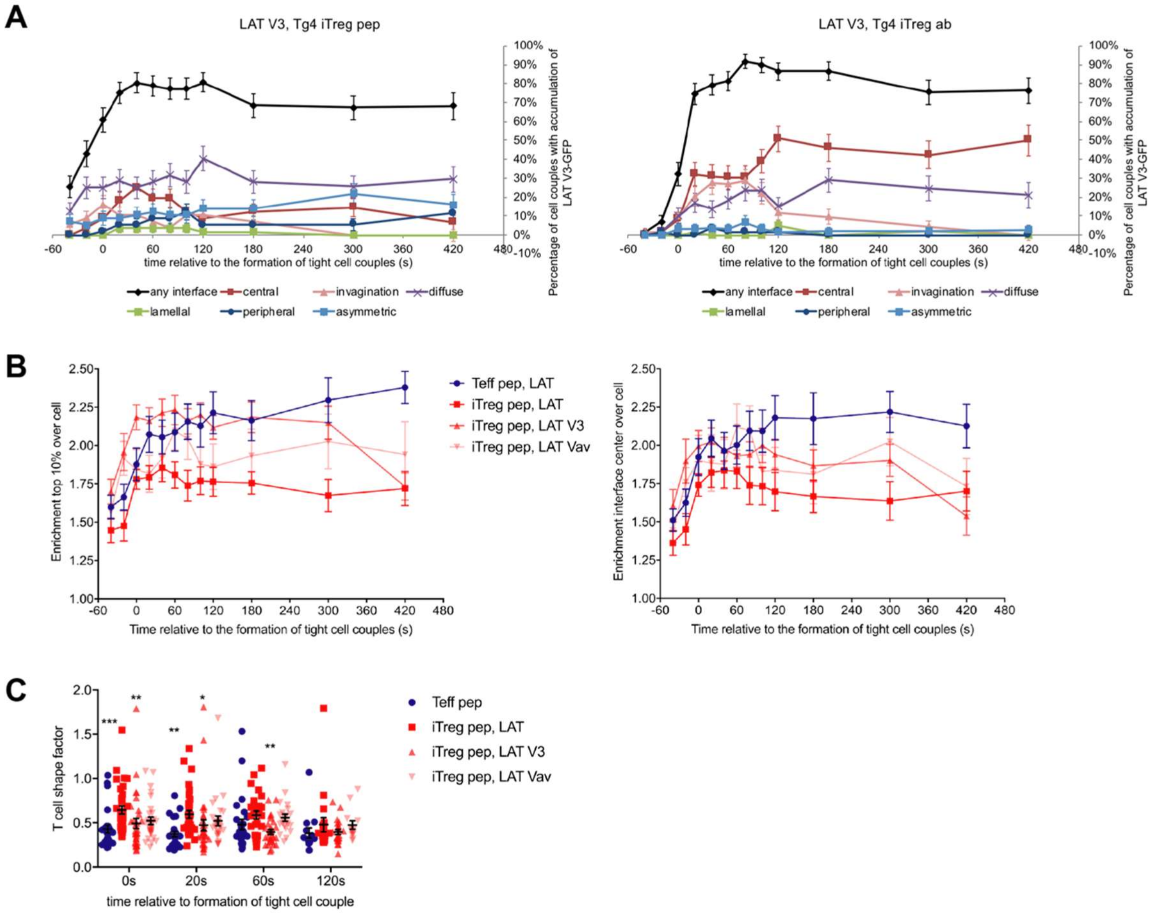

Figure S6. cSMAC formation can be partially restored with synthetic approaches in Tg4 iTreg pep cells. **A** Tg4 iTreg cells as indicated transduced to express LAT V3-GFP were activated with PL8 APCs (10 μg/ml Ac1-9[4Y]). Data are expressed as the percentage of cell couples with LAT V3-GFP accumulation in the indicated patterns (Supplementary Figure S2A) relative to tight cell couple formation. Number of cell couples analyzed is n=59 for iTreg ab cells from 4 independent experiments. iTreg pep data are from Figure 6B. Statistical significance of differences between conditions is given in Table S9. **B** Computational image analysis similar to Supplementary Figure S3 of the same data as in Figure 6B. On the left, data are expressed as enrichment in the 10% of the cell volume with the highest LAT-GFP, LAT-V3-GFP or LAT Vav-GFP intensity relative to the rest of the cell relative to tight cell couple formation. On the right, data are expressed as enrichment in the central interface cylinder relative to the rest of the cell relative to tight cell couple formation as indicated. LAT-GFP data are from Supplementary Figure S3. **C** Similar to Supplementary Figure S5C right, the same data as in Figure 6B are expressed as the T cell shape factor (ratio of lamellal length to interface diameter) at the indicated time relative to tight cell coupling. LAT-GFP data are from Supplementary Figure S5C.

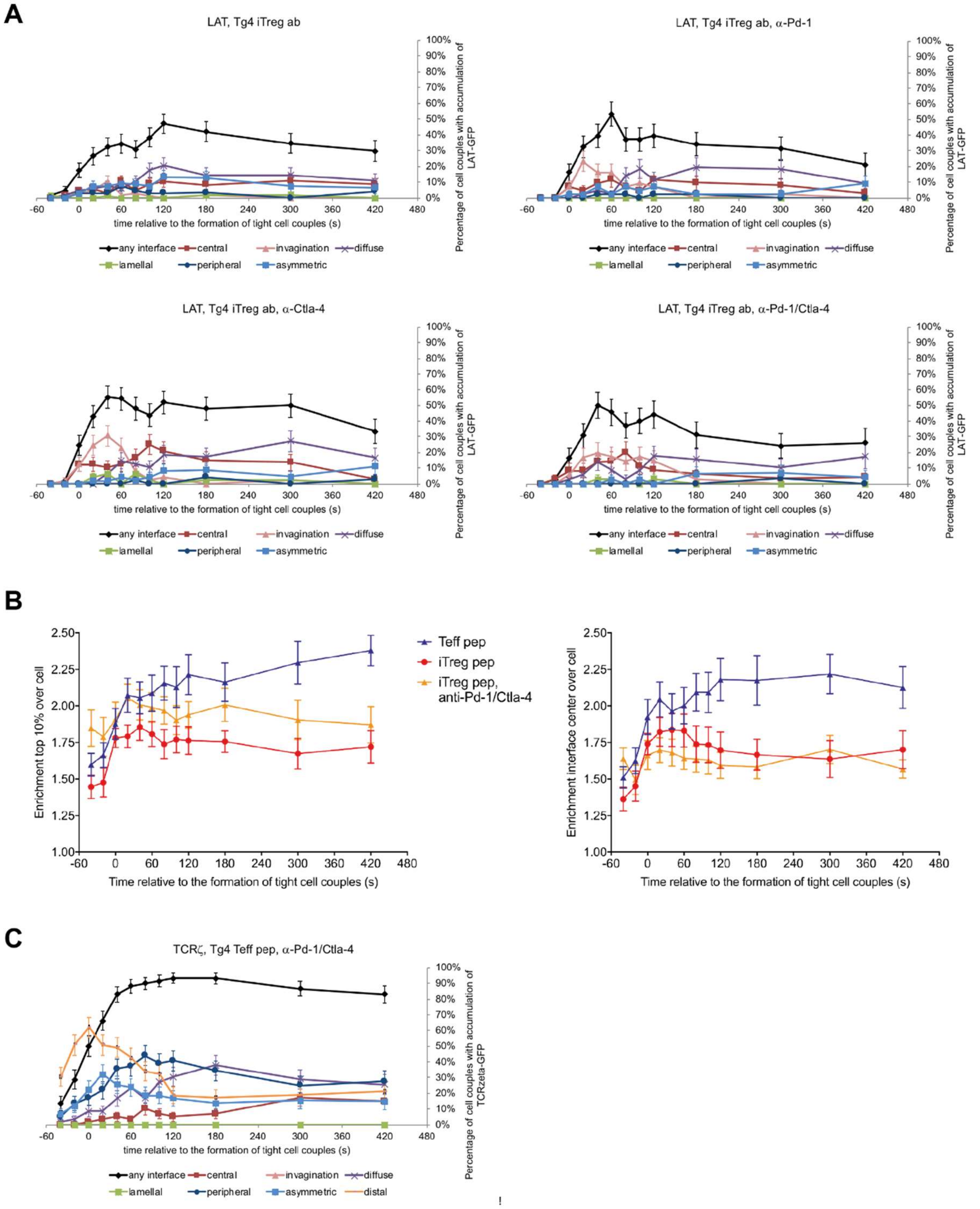

Figure S7. Pd-1 and Ctla-4 impair cSMAC formation upon induction of a regulatory phenotype. **A** Tg4 T cells as indicated transduced to express LAT-GFP were activated with PL8 APCs (10 μg/ml Ac1-9[4Y]). Data are expressed as the percentage of cell couples with LAT-GFP accumulation in the indicated patterns (Supplementary Figure S2A) relative to tight cell couple formation. Number of cell couples analyzed are n=43, 49, 37 for iTreg ab cells in the presence of 10 μg/ml α-Pd-1, 10 μg/ml α-Ctla-4 and 10 μg/ml α-Pd-1 plus α-Ctla-4, respectively, from 2-5 independent experiments each. Statistical significance of differences between conditions is given in Table S11. Tg4 iTreg ab buffer only data are from Supplementary Figure S2D. **B** Computational image analysis similar to Supplementary Figure S3 of the same data as in Figure 7B. On the left, data are expressed as enrichment in the 10% of the cell volume with the highest LAT-GFP intensity relative to the rest of the cell. On the right, data are expressed as enrichment in the central interface cylinder relative to the rest of the cell relative to tight cell couple formation. Tg4 Teff pep and iTreg pep data are from Supplementary Figure S3. **C** Tg4 T cells as indicated transduced to express TCRζ-GFP were activated with PL8 APCs (10 μg/ml Ac1-9[4Y]). The graphs display the percentage of cell couples with TCRζ-GFP accumulation in the indicated patterns (Supplementary Figure S2A) relative to tight cell couple. Number of cell couples analyzed are n=59 from 2 independent experiments..

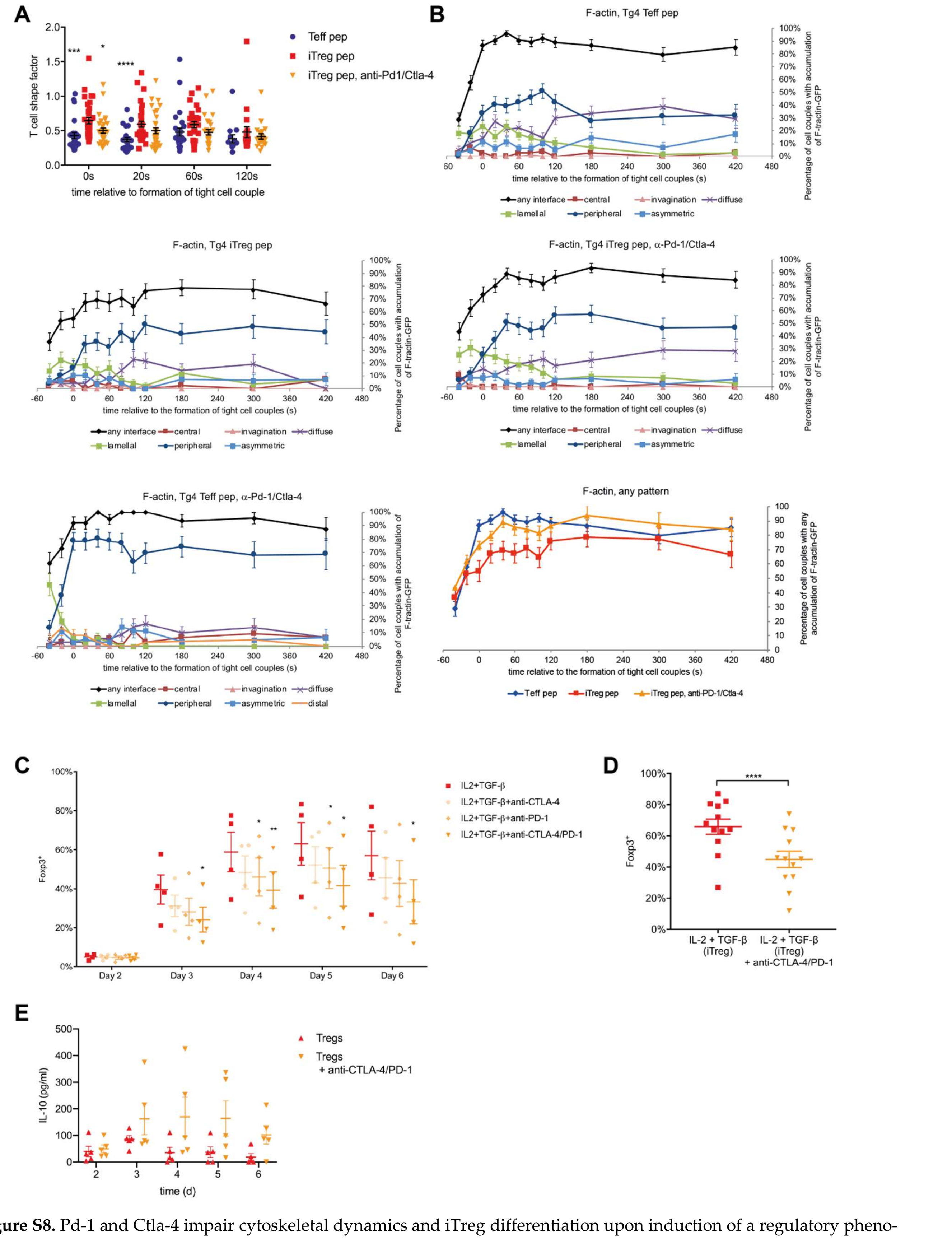

Figure S8. Pd-1 and Ctla-4 impair cytoskeletal dynamics and iTreg differentiation upon induction of a regulatory phenotype. **A** Tg4 T cells as indicated transduced to express LAT-GFP were activated with PL8 APCs (10 µg/ml Ac1-9[4Y]). These are the same cells as in Figure 7B. Similar to Supplementary Figure S5C right, data are expressed as the T cell shape factor (ratio of lamellal length to interface diameter) at the indicated time relative to tight cell coupling. Teff pep and iTreg pep data are from Supplementary Figure S5C. * $p<0.05$, *** $p<0.001$, **** $p<0.0001$ vs. iTreg pep at the same time. **B** Tg4 T

cells as indicated transduced to express F-tractin-GFP were activated with PL8 APCs (10 μg/ml Ac1-9[4Y]). The graphs display the percentage of cell couples with F-tractin-GFP accumulation in the indicated patterns (Supplementary Figure S2A) relative to tight cell couple. Number of cell couples analyzed are n=56, 37 for iTreg pep, Teff pep cells in the presence of 10 μg/ml α-Pd-1 plus α-Ctla-4 from 3-5 independent experiments. Statistical significance of differences between conditions is given in Table S10. Control Teff pep and iTreg pep data are from Figure 5B. The panel in row three at the right is a direct comparison of accumulation in any pattern under the indicated three experimental conditions. **C, D** Tg4 splenocytes were incubated with 10 μg/ml MBP Ac1-9[4K] peptide for 6 days in the presence of 10 ng/ml TGFβ plus 100 U/ml IL-2 upon addition of 10 μg/ml α-Pd-1, 10 μg/ml α-Ctla-4, 10 μg/ml α-Pd-1 plus α-Ctla-4 or buffer only. C Data are expressed as the percentage FoxP3+ cells (n=4 independent experiments). * p<0.05, ** p<0.01 vs. buffer only on the same day. D Data from a larger number of experiments including those in E are expressed as the percentage FoxP3+ cells on day 6 only. The part of the data without antibody treatment are the same as Figure 1B. (n=12, 12 independent experiments). **** p<0.0001. **E** Tg4 splenocytes were incubated with 10 μg/ml MBP Ac1-9[4K] peptide for 6 days in the presence of 10 ng/ml TGFβ plus 100 U/ml IL-2 upon addition of 10 μg/ml α-Pd-1 plus α-Ctla-4 or buffer only and IL-10 amounts in tissue culture supernatants were determined by ELISA. Data are expressed as amount of cytokine (n=4-5 independent experiments). Data points are slightly nudged to increase legibility. The difference between α-Pd-1 plus α-Ctla-4 and buffer only is significant with p<0.01 by 2-way ANOVA.

Table S1. Statistical analysis of Figure 2B.

Condition	Comparison	Pattern	-40	-20	0	20	40	60	80	100	120	180	300	420
LAT, Teff pep	LAT, iTreg pep	any			0.02	0.000	0.000	0.04	0.03	0.03		0.02		0.04
		central			0.000	0.000	0.000	0.007	0.007		0.007			
		invagination												
LAT, Teff pep	LAT, Teff 9608	any				0.005								
		central												
		invagination				0.02								
LAT, Teff pep	LAT, Ttol	any									0.04		0.03	0.04
		central				0.003	0.02	0.01	0.02		0.03			
		invagination												

Table S2. Statistical analysis of Supplementary Figure S2D.

Condition	Comparison	Pattern	-40	-20	0	20	40	60	80	100	120	180	300	420
LAT, Teff ab	LAT, iTreg ab	any			0.001	0.001	0.002	0.001	0.000	0.001		0.000	0.02	0.004
		central			0.01		0.005		0.001	0.03	0.03			
		invagination			0.03	0.001	0.007	0.001	0.01	0.02				
LAT, Teff ab	LAT, Teff pep	any							0.01	0.01	0.02	0.000	0.002	0.002
		central				0.04								
		invagination												
LAT, iTreg ab	LAT, iTreg pep	any									0.008	0.000	0.01	0.01
		central												
		invagination				0.02		0.006						

Table S3. Statistical analysis of Figure 3A, B.

Condition	Comparison	Pattern	-40	-20	0	20	40	60	80	100	120	180	300	420
DAG, Teff pep	DAG, Ttol	any					0.02	0.001	0.02	0.03	0.006	0.03		
		central												
TCRζ Teff pep	TCRζ, Ttol	any												0.004
		central											0.03	0.03
		distal				0.05	0.008	0.005	0.05					
	TCRζ, iTreg pep	any	0.002	0.002	0.02	0.002							0.02	0.02
		central												0.02
		distal				0.006	0.005						0.008	0.00[illegible]

Table S4. Statistical analysis of Supplementary Figure S4.

Condition	Comparison	Pattern	-40	-20	0	20	40	60	80	100	120	180	300	420
TCRζ, iTreg pep	TCRζ, iTreg ab	any	0.004	0.000	0.000	0.001	0.002							0.006
		central												
		distal			0.04	0.04						0.02	0.02	

Table S5. Statistical analysis of Figure 5B.

Condition	Comparison	Pattern	-40	-20	0	20	40	60	80	100	120	180	300	420
F-actin, Teff pep	F-actin, iTreg pep	any			0.000	0.002	0.000	0.002	0.02	0.000				
		peripheral			0.05									
		lamellal												
		lamellal + diffuse				0.03	0.04							0.04
F-actin, Teff pep	F-actin, Teff 9608	any	0.003	0.004	0.000		0.001						0.02	0.04
		peripheral				0.04						0.02		
		lamellal					0.03							
		lamellal + diffuse	0.01		0.01	0.001	0.004	0.04	0.04		0.005	0.01	0.02	
F-actin, Teff pep	F-actin, Ttol	any			0.01									
		peripheral						0.001		0.02	0.02	0.001		
		lamellal					0.03							
		lamellal + diffuse			0.03	0.000	0.000	0.000	0.007	0.006	0.000	0.000	0.002	0.008

Table S6. Statistical analysis of Supplementary Figure S5A.

Condition	Comparison	Pattern	-40	-20	0	20	40	60	80	100	120	180	300	420
F-actin, iTreg pep	F-actin, iTreg ab	any		0.04										
		peripheral							0.05					
		lamellal				0.02								

Table S7. Statistical analysis of Figure 6B.

Condition	Comparison	Pattern	-40	-20	0	20	40	60	80	100	120	180	300	420
LAT, iTreg pep	LAT V3, iTreg pep	any	0.000	0.000	0.000	0.000	0.000	0.000	0.000	0.000	0.000	0.000	0.000	0.000
		central				0.05	0.001	0.03					0.05	
		invagination												
LAT, iTreg pep	LAT Vav, iTreg pep	any					0.005	0.001	0.002	0.003	0.01	0.000	0.000	0.002
		central					0.05		0.05					
		invagination												
LAT, Teff pep	LAT V3, iTreg pep	any	0.007	0.000	0.05			0.005	0.005	0.001	0.000	0.000	0.000	0.000
		central			0.009	0.01					0.05			
		invagination												
LAT, Teff pep	LAT Vav, iTreg pep	any											0.03	
		central			0.02	0.02								
		invagination												

Table S8. Statistical analysis of Supplementary Figure S6A.

Condition	Comparison	Pattern	-40	-20	0	20	40	60	80	100	120	180	300	420
LAT V3, iTreg pep	LAT V3, iTreg ab	any												
		central								0.002	0.000	0.000	0.004	0.000
		invagination					0.04	0.009	0.001					

Table S9. Statistical analysis of Figure 7B, D, Supplementary Figure S8.

Condition	Comparison	Pattern	-40	-20	0	20	40	60	80	100	120	180	300	420
LAT, iTreg pep, α-Pd-1/Ctla-4	LAT Teff pep	any				0.01	0.03							
		central				0.000	0.03							
		invagination												
	LAT iTreg pep	any			0.007							0.02	0.03	0.01
		central			0.03									
		invagination												
F-tractin, iTreg pep, α-Pd-1/Ctla-4	F-tractin Teff pep	any												
		peripheral										0.003		
		lamellal												
		Lamellal + difuse												
	F-tractin iTreg pep	any					0.02	0.05						
		peripheral												
		lamellal												
		Lamellal + difuse												
TCRζ, iTreg pep, α-Pd-1/Ctla-4	TCRζ, Teff pep	any	0.01	0.000	0.000	0.000	0.001	0.004	0.004	0.003	0.008	0.000		
		central												
		distal					0.05	0.04		0.04				
	TCRζ, iTreg pep	any			0.001	0.007	0.04	0.000	0.000	0.000	0.000	0.000	0.000	0.000
		central							0.02			0.000	0.000	0.000
		distal											0.05	0.01

Table S10. Statistical analysis of Supplementary Figure S7A.

Condition	Comparison	Pattern	-40	-20	0	20	40	60	80	100	120	180	300	420
LAT, iTreg ab	LAT, iTreg ab, α-Pd-1	any												
		central												
		invagination				0.007		0.01						
LAT, iTreg ab	LAT, iTreg ab, α-Ctla-4	any					0.02	0.05						
		central								0.03				
		invagination				0.003	0.01	0.001						
LAT, iTreg ab	LAT, iTreg ab, α-Pd-1/Ctla-4	any												
		central							0.02					
		invagination						0.01		0.001	0.006			

Video S1. A representative interaction of a Tg4 T eff pep cell retrovirally transduced to express LAT-GFP with a PL8 B cell lymphoma APCs and 10 μg/ml MBP Ac1-9[4Y] peptide is shown. DIC images are shown on the top, with matching top-down, maximum projections of 3D sensor fluorescence data on the bottom. The sensor fluorescence intensity is displayed in a rainbow-like, false-color scale (increasing from blue to red). 20 s intervals in video acquisition are played back as 2 frames per second. Cell coupling occurs in frame 4 (2s indicated video time).

Video S2. The video is displayed similar to Video S1. A Tg4 Ttol cell is transduced to express LAT-GFP and activated as in Video S1. Cell coupling occurs in frame 3 (1s indicated video time).

Video S3. The video is displayed similar to Video S1. A Tg4 iTreg cell is transduced to express LAT-GFP and activated as in Video S1. Cell coupling occurs in frame 5 (2s indicated video time).

Video S4. The video is displayed similar to Video S1. A Tg4 Teff iPI4K cell is transduced to express LAT-GFP and activated as in Video S1. Cell coupling occurs in frame 7 (3s indicated video time).

Video S5. The video is displayed similar to Video S1. A Tg4 T eff pep cell is transduced to express tandem C1 domain-GFP and activated as in Video S1. Cell coupling occurs in frame 6 (3s indicated video time).

Video S6. The video is displayed similar to Video S1. A Tg4 T eff pep cell is transduced to express TCRζ-GFP and activated as in Video S1. Cell coupling occurs in frame 6 (3s indicated video time).

Video S7. The video is displayed similar to Video S1. A Tg4 T eff pep cell is transduced to express F-tractin-GFP and activated as in Video S1. Cell coupling occurs in frame 7 (3s indicated video time).

Video S8. The video is displayed similar to Video S1. A Tg4 iTreg cell is transduced to express LAT V3-GFP and activated as in Video S1. Cell coupling occurs in frame 7 (3s indicated video time).

Video S9. The video is displayed similar to Video S1. A Tg4 iTreg cell is transduced to express LAT-GFP and activated as in Video S1 upon addition of 10 μg/ml α-Pd-1 plus α-Ctla-4. Cell coupling occurs in frame 8 (4s indicated video time).
