# Supplementary material for: A LAT-Based Signaling Complex in the Immunological Synapse as Determined with Live Cell Imaging Is Less Stable in T Cells with Regulatory Capability
Source: Cells. 2021 Feb 17;10(2):418. doi: 10.3390/cells10020418 (PMC7921939; doi:10.3390/cells10020418)
Supplement: Supplementary file 1 [file cells-10-00418-s001.zip › supplement/table S2.docx]

| **Condition** | **Comparison** | **Pattern** | **-40** | **-20** | **0** | **20** | **40** | **60** | **80** | **100** | **120** | **180** | **300** | **420** |  | **early** | **late** |
| --- | --- | --- | --- | --- | --- | --- | --- | --- | --- | --- | --- | --- | --- | --- | --- | --- | --- |
|  |  |  |  |  |  |  |  |  |  |  |  |  |  |  |  |  |  |
| LAT, Teff ab | LAT, iTreg ab | any |  |  | 0.001 | 0.001 | 0.002 | 0.001 | 0.000 | 0.001 |  | 0.000 | 0.02 | 0.004 |  | 0.000 | 0.000 |
|  |  | central |  |  | 0.01 |  | 0.005 |  | 0.001 | 0.03 | 0.03 |  |  |  |  | 0.000 | 0.001 |
|  |  | invagination |  |  | 0.03 | 0.001 | 0.007 | 0.001 | 0.01 | 0.02 |  |  |  |  |  | 0.000 |  |
|  |  |  |  |  |  |  |  |  |  |  |  |  |  |  |  |  |  |
| LAT, Teff ab | LAT, Teff pep | any |  |  |  |  |  |  | 0.01 | 0.01 | 0.02 | 0.000 | 0.002 | 0.002 |  | 0.002 | 0.000 |
|  |  | central |  |  |  | 0.04 |  |  |  |  |  |  |  |  |  |  |  |
|  |  | invagination |  |  |  |  |  |  |  |  |  |  |  |  |  | 0.009 |  |
|  |  |  |  |  |  |  |  |  |  |  |  |  |  |  |  |  |  |
| LAT, iTreg ab | LAT, iTreg pep | any |  |  |  |  |  |  |  |  | 0.008 | 0.000 | 0.01 | 0.01 |  |  | 0.000 |
|  |  | central |  |  |  |  |  |  |  |  |  |  |  |  |  |  | 0.02 |
|  |  | invagination |  |  |  | 0.02 |  | 0.006 |  |  |  |  |  |  |  | 0.000 |  |
