# Supplementary material for: A LAT-Based Signaling Complex in the Immunological Synapse as Determined with Live Cell Imaging Is Less Stable in T Cells with Regulatory Capability
Source: Cells. 2021 Feb 17;10(2):418. doi: 10.3390/cells10020418 (PMC7921939; doi:10.3390/cells10020418)
Supplement: Supplementary file 1 [file cells-10-00418-s001.zip › supplement/table S3.docx]

| Condition | Comparison | Pattern | -40 | -20 | 0 | 20 | 40 | 60 | 80 | 100 | 120 | 180 | 300 | 420 |  | early | late |
| --- | --- | --- | --- | --- | --- | --- | --- | --- | --- | --- | --- | --- | --- | --- | --- | --- | --- |
|  |  |  |  |  |  |  |  |  |  |  |  |  |  |  |  |  |  |
| DAG, Teff pep | DAG, Ttol | any |  |  |  |  | 0.02 | 0.001 | 0.02 | 0.03 | 0.006 | 0.03 |  |  |  | 0.000 | 0.000 |
|  |  | central |  |  |  |  |  |  |  |  |  |  |  |  |  |  |  |
|  |  |  |  |  |  |  |  |  |  |  |  |  |  |  |  |  |  |
|  |  |  |  |  |  |  |  |  |  |  |  |  |  |  |  |  |  |
| TCRζ Teff pep | TCRζ, Ttol | any |  |  |  |  |  |  |  |  |  |  |  | 0.004 |  |  | 0.000 |
|  |  | central |  |  |  |  |  |  |  |  |  |  | 0.03 | 0.03 |  | 0.004 | 0.000 |
|  |  | distal |  |  |  | 0.05 | 0.008 | 0.005 | 0.05 |  |  |  |  |  |  | 0.000 |  |
|  |  |  |  |  |  |  |  |  |  |  |  |  |  |  |  |  |  |
|  | TCRζ, iTreg pep | any | 0.002 | 0.002 | 0.02 | 0.002 |  |  |  |  |  |  | 0.02 | 0.02 |  | 0.05 | 0.000 |
|  |  | central |  |  |  |  |  |  |  |  |  |  |  | 0.02 |  | 0.001 | 0.000 |
|  |  | distal |  |  |  | 0.006 | 0.005 |  |  |  |  |  | 0.008 | 0.000 |  | 0.000 | 0.004 |
|  |  |  |  |  |  |  |  |  |  |  |  |  |  |  |  |  |  |
|  |  |  |  |  |  |  |  |  |  |  |  |  |  |  |  |  |  |
