# Supplementary material for: A LAT-Based Signaling Complex in the Immunological Synapse as Determined with Live Cell Imaging Is Less Stable in T Cells with Regulatory Capability
Source: Cells. 2021 Feb 17;10(2):418. doi: 10.3390/cells10020418 (PMC7921939; doi:10.3390/cells10020418)
Supplement: Supplementary file 1 [file cells-10-00418-s001.zip › supplement/table S9.docx]

| Condition | Comparison | Pattern | -40 | -20 | 0 | 20 | 40 | 60 | 80 | 100 | 120 | 180 | 300 | 420 |  | early | late |
| --- | --- | --- | --- | --- | --- | --- | --- | --- | --- | --- | --- | --- | --- | --- | --- | --- | --- |
|  |  |  |  |  |  |  |  |  |  |  |  |  |  |  |  |  |  |
| LAT, iTreg pep, α-Pd-1/Ctla-4 | LAT Teff pep | any |  |  |  | 0.01 | 0.03 |  |  |  |  |  |  |  |  | 0.001 |  |
|  |  | central |  |  |  | 0.000 | 0.03 |  |  |  |  |  |  |  |  | 0.000 | 0.04 |
|  |  | invagination |  |  |  |  |  |  |  |  |  |  |  |  |  |  |  |
|  |  |  |  |  |  |  |  |  |  |  |  |  |  |  |  |  |  |
|  | LAT iTreg pep | any |  |  | 0.007 |  |  |  |  |  |  | 0.02 | 0.03 | 0.01 |  | 0.001 | 0.000 |
|  |  | central |  |  | 0.03 |  |  |  |  |  |  |  |  |  |  | 0.001 |  |
|  |  | invagination |  |  |  |  |  |  |  |  |  |  |  |  |  |  |  |
|  |  |  |  |  |  |  |  |  |  |  |  |  |  |  |  |  |  |
| F-tractin, iTreg pep, α-Pd-1/Ctla-4 | F-tractin Teff pep | any |  |  |  |  |  |  |  |  |  |  |  |  |  | 0.000 |  |
|  |  | peripheral |  |  |  |  |  |  |  |  |  | 0.003 |  |  |  |  | 0.000 |
|  |  | lamellal |  |  |  |  |  |  |  |  |  |  |  |  |  |  |  |
|  |  | Lamellal + difuse |  |  |  |  |  |  |  |  |  |  |  |  |  |  | 0.04 |
|  |  |  |  |  |  |  |  |  |  |  |  |  |  |  |  |  |  |
|  | F-tractin iTreg pep | any |  |  |  |  | 0.02 | 0.05 |  |  |  |  |  |  |  |  | 0.004 |
|  |  | peripheral |  |  |  |  |  |  |  |  |  |  |  |  |  | 0.05 |  |
|  |  | lamellal |  |  |  |  |  |  |  |  |  |  |  |  |  | 0.04 |  |
|  |  | Lamellal + difuse |  |  |  |  |  |  |  |  |  |  |  |  |  | 0.001 |  |
|  |  |  |  |  |  |  |  |  |  |  |  |  |  |  |  |  |  |
| TCRζ, iTreg pep, α-Pd-1/Ctla-4 | TCRζ, Teff pep | any | 0.01 | 0.000 | 0.000 | 0.000 | 0.001 | 0.004 | 0.004 | 0.003 | 0.008 | 0.000 |  |  |  | 0.000 | 0.000 |
|  |  | central |  |  |  |  |  |  |  |  |  |  |  |  |  |  | 0.002 |
|  |  | distal |  |  |  |  | 0.05 | 0.04 |  | 0.04 |  |  |  |  |  | 0.000 |  |
|  |  |  |  |  |  |  |  |  |  |  |  |  |  |  |  |  |  |
|  | TCRζ, iTreg pep | any |  |  | 0.001 | 0.007 | 0.04 | 0.000 | 0.000 | 0.000 | 0.000 | 0.000 | 0.000 | 0.000 |  | 0.000 | 0.000 |
|  |  | central |  |  |  |  |  |  | 0.02 |  |  | 0.000 | 0.000 | 0.000 |  | 0.000 | 0.000 |
|  |  | distal |  |  |  |  |  |  |  |  |  |  | 0.05 | 0.01 |  |  | 0.000 |
