# Supplementary figures and images for: A LAT-Based Signaling Complex in the Immunological Synapse as Determined with Live Cell Imaging Is Less Stable in T Cells with Regulatory Capability
Source: Cells. 2021 Feb 17;10(2):418. doi: 10.3390/cells10020418 (PMC7921939; doi:10.3390/cells10020418)

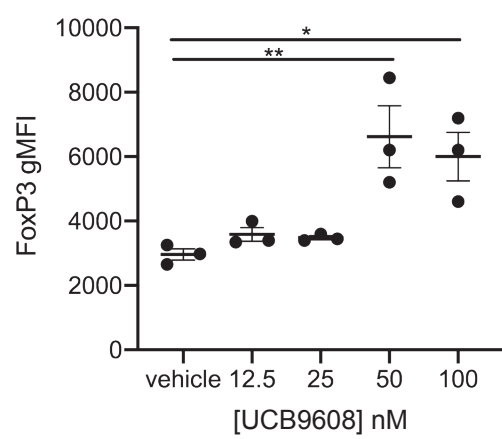

Supplement: Supplementary file 1 [file cells-10-00418-s001.zip › supplement/Fig. S1.pdf]

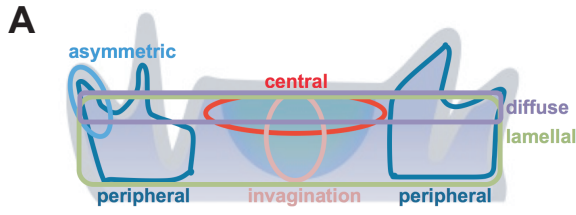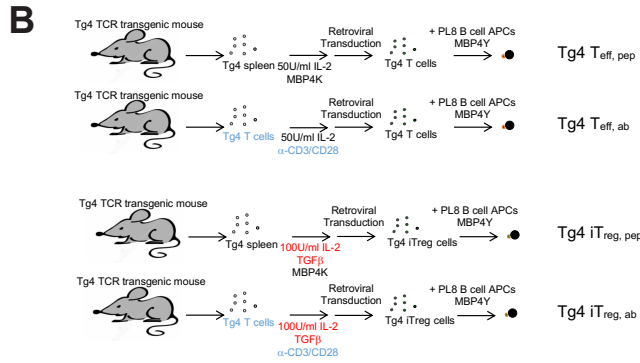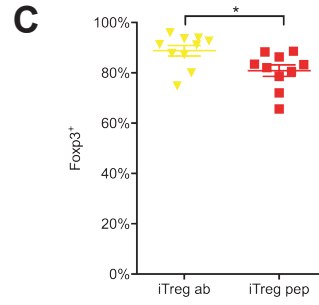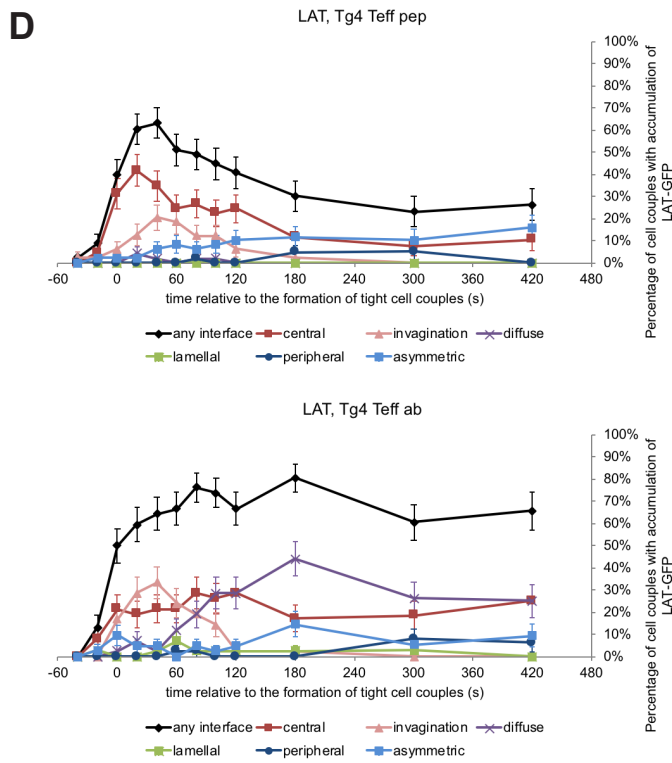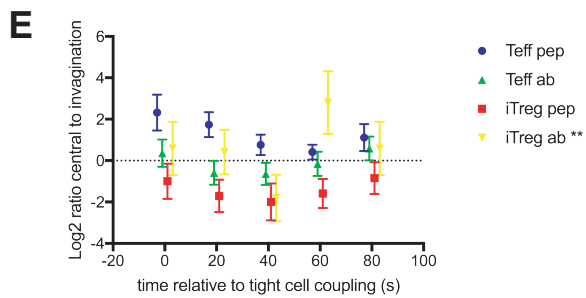

Supplement: Supplementary file 1 [file cells-10-00418-s001.zip › supplement/Fig. S2.pdf]

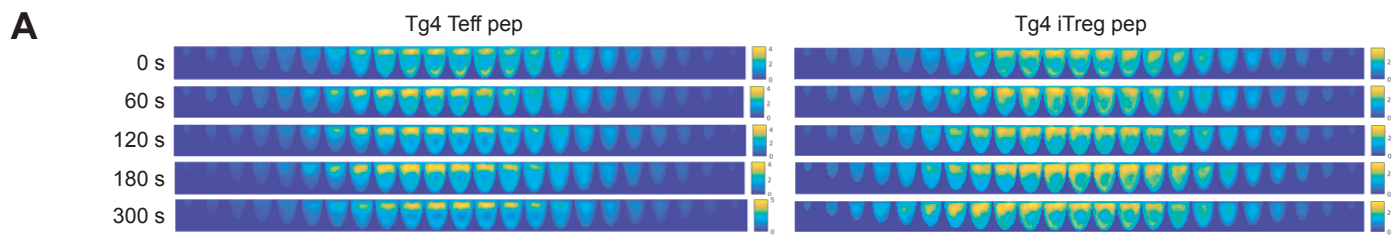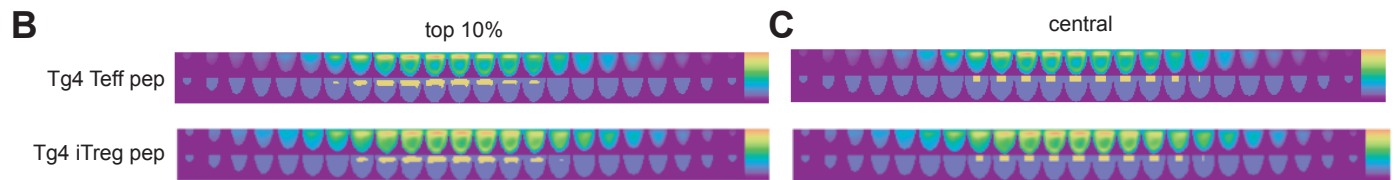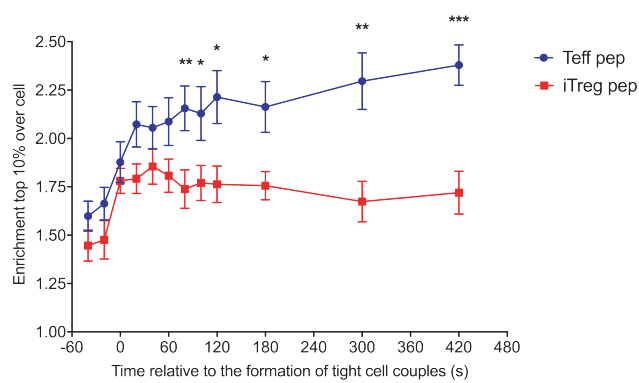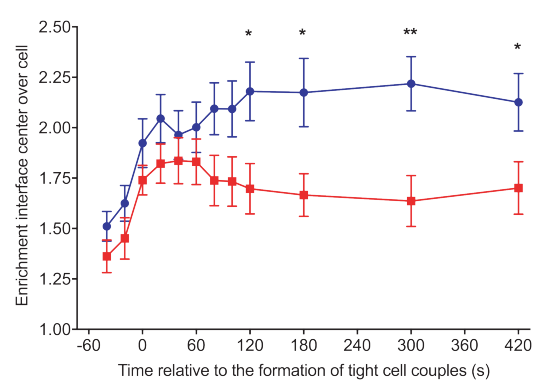

Supplement: Supplementary file 1 [file cells-10-00418-s001.zip › supplement/Fig. S3.pdf]

TCR $\zeta$ , Tg4 iTreg pep

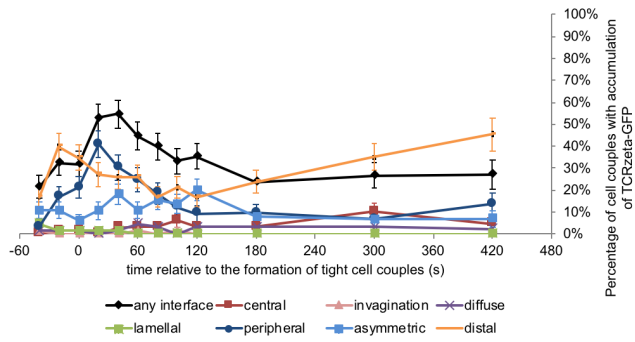

TCR $\zeta$ , Tg4 iTreg ab

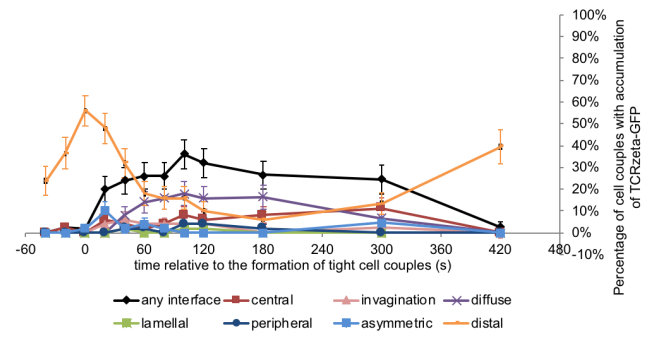

Supplement: Supplementary file 1 [file cells-10-00418-s001.zip › supplement/Fig. S4.pdf]

**A**

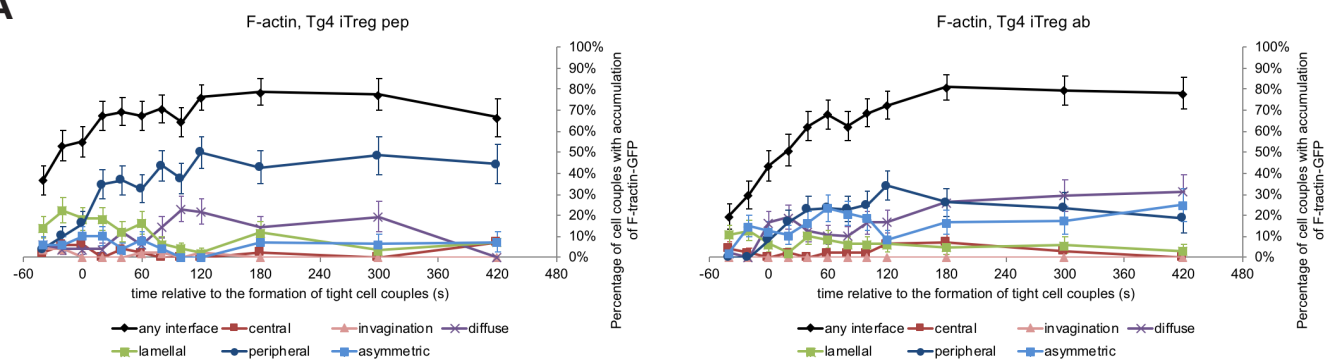

**B**

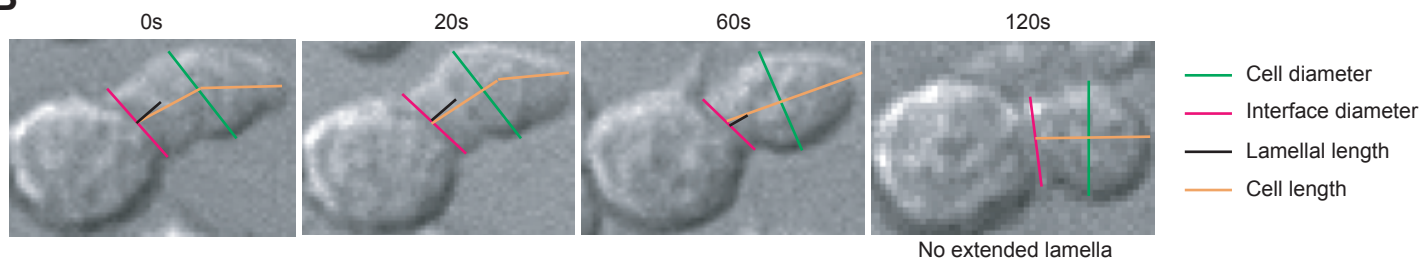

**C**

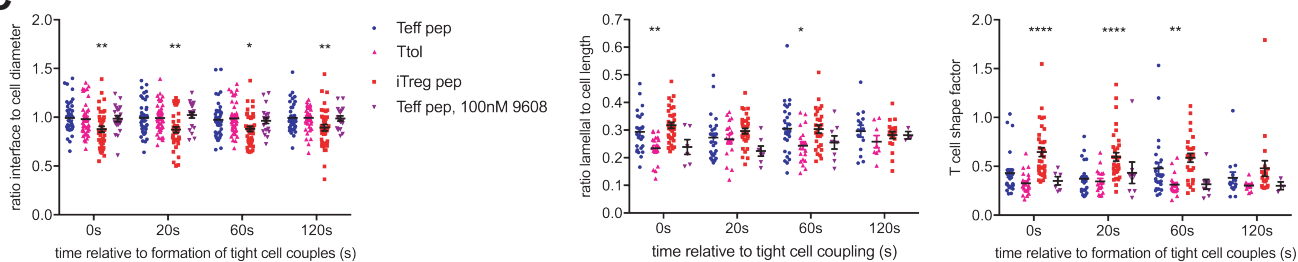

**D**

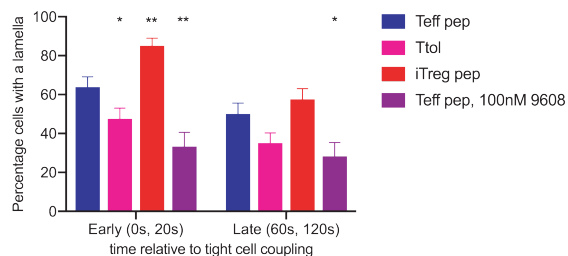

Supplement: Supplementary file 1 [file cells-10-00418-s001.zip › supplement/Fig. S5.pdf]

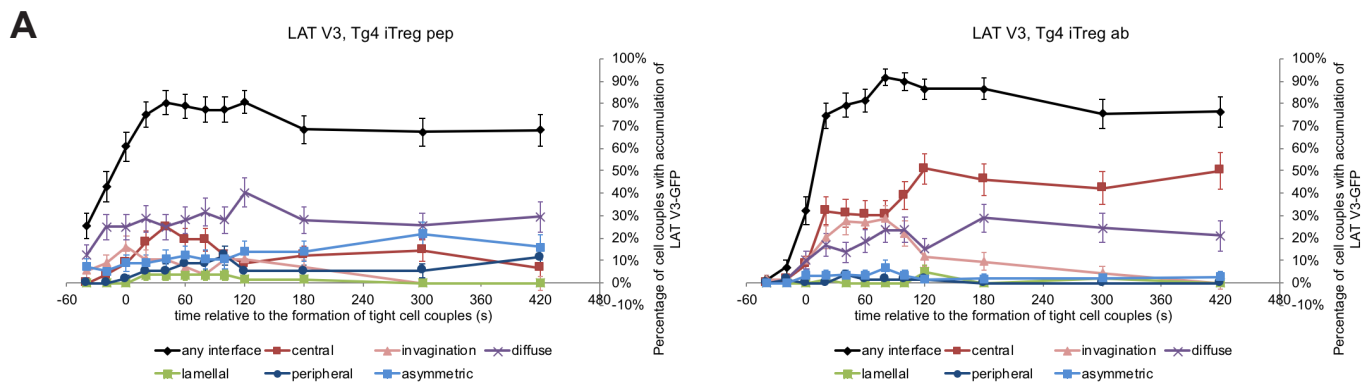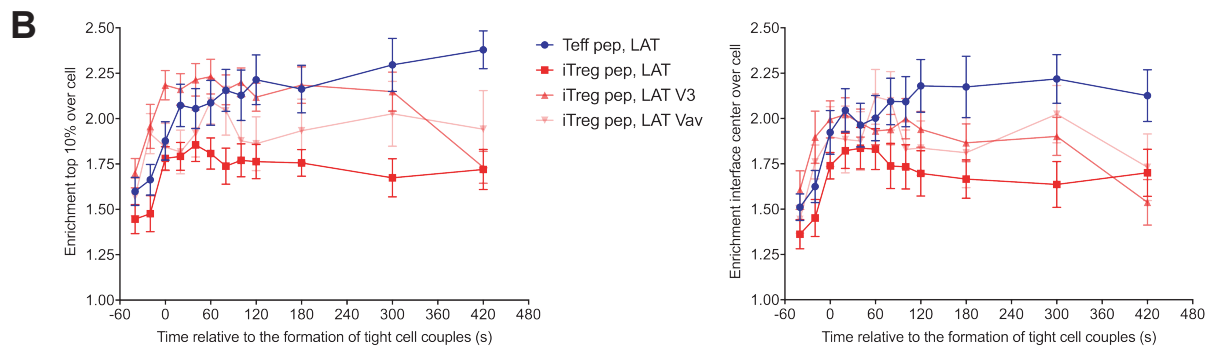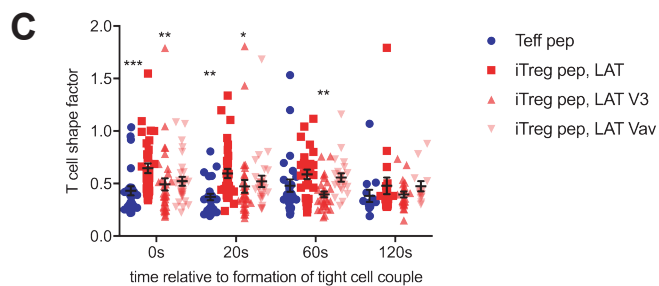

Supplement: Supplementary file 1 [file cells-10-00418-s001.zip › supplement/Fig. S6.pdf]

**A**

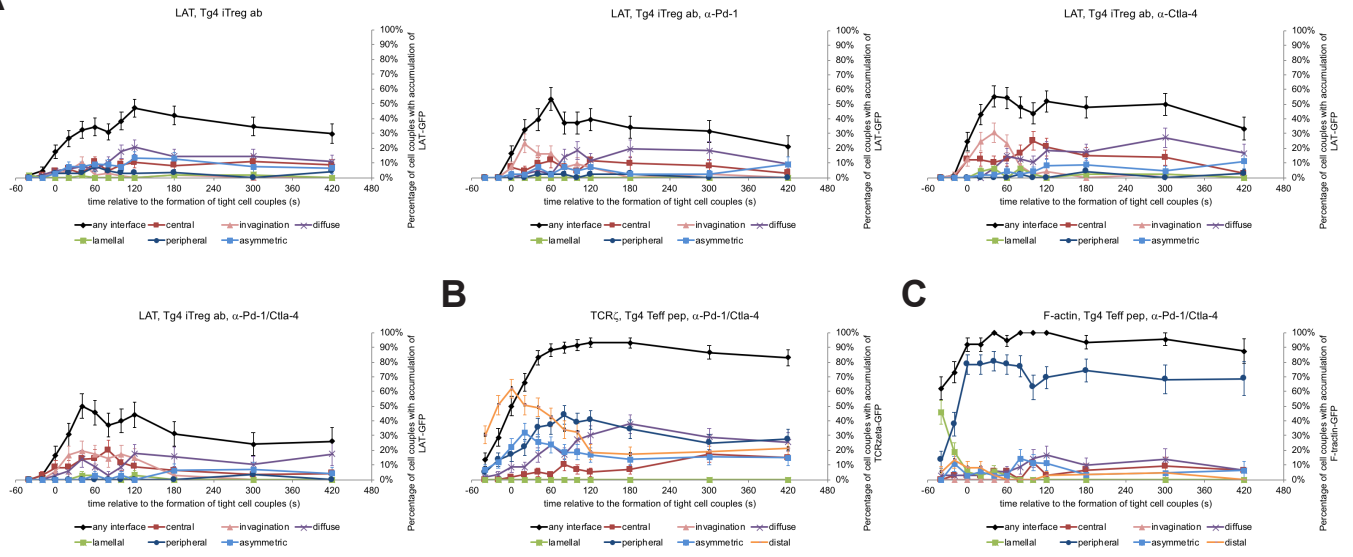

D

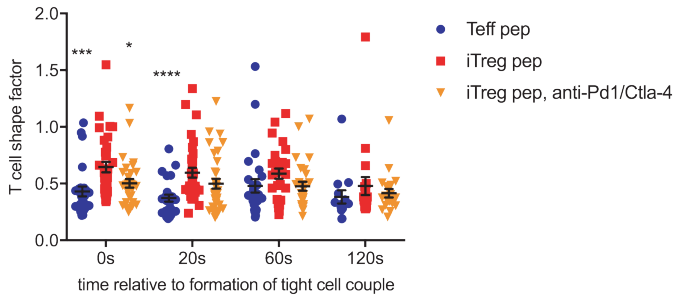

# E

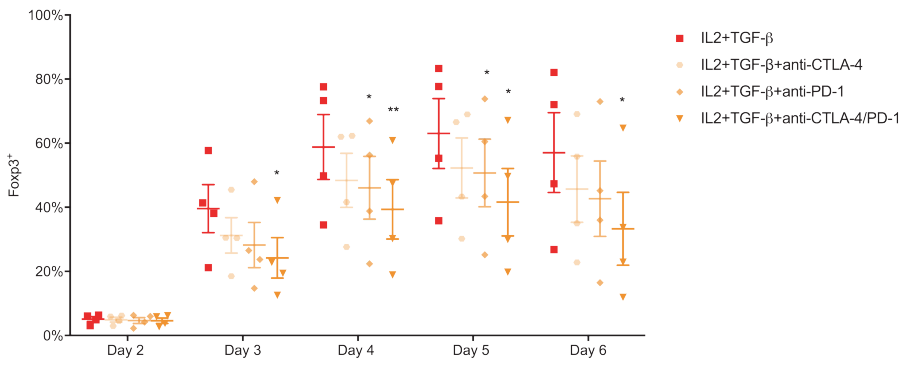**F**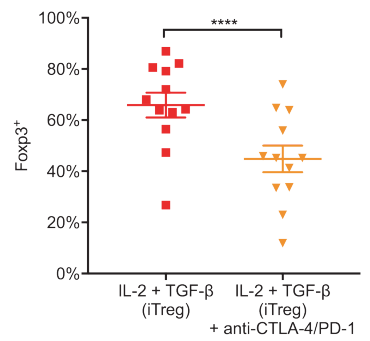

## G

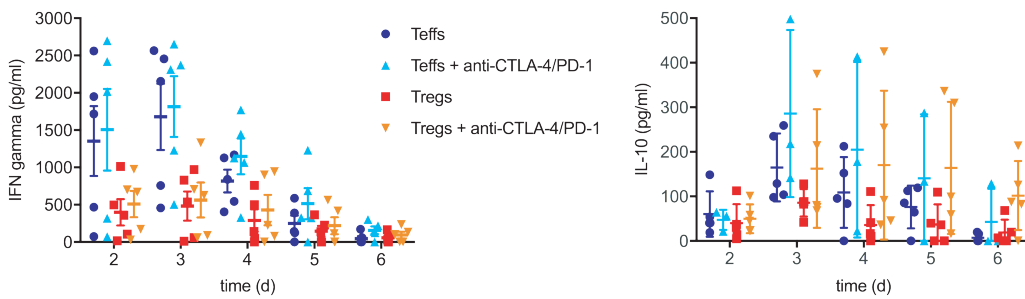

Supplement: Supplementary file 1 [file cells-10-00418-s001.zip › supplement/Fig. S7.pdf]

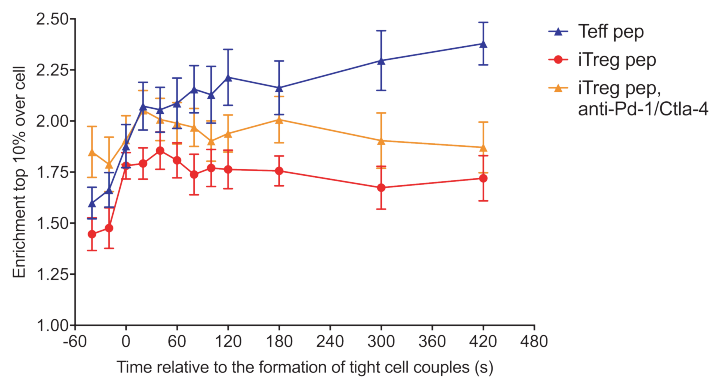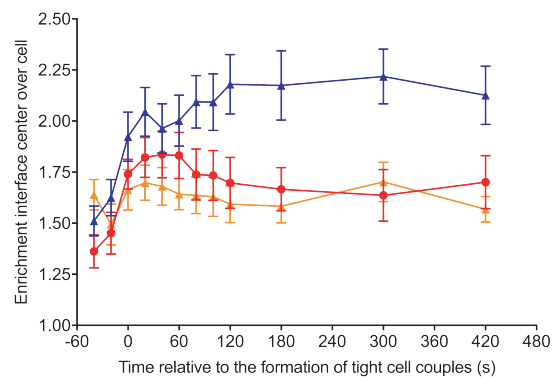

Supplement: Supplementary file 1 [file cells-10-00418-s001.zip › supplement/Fig. S8.pdf]
